# SRSF3 Expression Serves as a Potential Biomarker for Prognostic and Immune Response in Pan-Cancer

**DOI:** 10.3389/fonc.2022.808530

**Published:** 2022-04-14

**Authors:** Zihua Li, Hui Huang, Xinbo Wu, Tao Yu, Fajiao Xiao, Haichao Zhou, Anquan Shang, Yunfeng Yang

**Affiliations:** ^1^ Department of Orthopedics, Shanghai Tongji Hospital, School of Medicine, Tongji University, Shanghai, China; ^2^ Department of Orthopedics, Shanghai Tenth People’s Hospital, School of Medicine, Tongji University, Shanghai, China; ^3^ Department of Laboratory Medicine, Shanghai Tongji Hospital, School of Medicine, Tongji University, Shanghai, China

**Keywords:** SRSF3, prognostic biomarkers, pan-cancer, TCGA, overall survival

## Abstract

Serine-rich splicing factor3 (SRSF3) plays an essential role in cell proliferation and inducing and maintaining of cancers as a proto-oncogene. However, the mechanisms of SRSF3 in pan-cancers are still unknown. In our study, a visualized prognostic landscape of SRSF3 in pan-cancer was investigated and the relationship between SRSF3 expression and immune infiltration was also investigated. The expression pattern and prognostic worth of SRSF3 among pan-cancers were explored through different databases, namely, the TCGA and Kaplan–Meier Plotter. Moreover, the survival analysis including Kaplan-Meier method for evaluating between groups was conducted. Further analyses including the correlation between expression SRSF expression and immune infiltration including tumor mutation burden (TMB), microsatellite instability (MSI) was investigated using Spearman test. In ACC, KIRP and UCEC cancer, upregulated expression of SRSF3 was associated with worse disease-free interval (DFI), representing a mechanism in promoting progression of tumor. Our results showed that SRSF3 expression was positively correlated immune cell infiltration, TMB, MSI in certain cancer types, indicating SRSF3 expression to potential value of therapy response. Additionally, we explored the functional characteristics of SRSF *in vitro* through western blot detecting the expression level of the apoptosis-related proteins in SW480 and 786-O cells. SRSF3 expression was upregulated in pan-cancer tissue compared with normal tissue, which confirmed by immunohistochemistry and its expression indicated poor overall survival and death-specific survival. Therefore, SRSF3 was found to be a possible biomarker for prognostic and therapeutic assessment through bioinformatic analysis. SRSF3 is expressed in various cancers and its high expression correlated to poor survival and disease progression. In summary, SRSF3 expression can be considered as a prognostic biomarker in pan-cancer and therapeutic evaluation.

## Introduction

The serine/arginine-rich splicing factors 1-12 (SRSF1-12) were structurally related to RNA-binding proteins, mediating interactions with proteins for regulating both constitutive and alternative splicing of pre-mRNA (1, 2). Additionally, SRSF members play essential roles in the regulation of transcription and post-splicing processes, which were imperative for cell cycle control. Cell cycle is closely related with the development and proliferation of malignant tumor cells. Increasing numbers of research have proposed that the malignant tumor behavior and its process can be altered by regulated cell cycle through genes. For instance, Guo et al. presented that overexpression of KAI1 could inhibit the proliferation in nasopharyngeal carcinoma cells (3). Additionally, Wu et al. and Zhang et al. found that PRC1 and aspirin alter the proliferation and cancer development through regulating cell cycle in oral squamous cell carcinoma (4, 5). Since the cell cycle has become an increasingly popular research direction, this study will extensively screen out genes that are related to the cell cycle in pan cancer, providing different prospectives and directions to treat cancer.

SRSF3, located on chromosome 6p21.31, is one of the smallest members of serine-rich protein family and its functions are promoting RNA splicing through recruiting components of the spliceosome at essential and alternatively spliced exon, which is essential for development (6–9). It has been reported in diverse cellular functions such as regulating cellular proliferation for controlling during G1/S through E2F transcription factor and also G2/M transition of immortal cell line of rats (10–12). These finding indicated that SRSF3 plays a crucial part as a proto-oncogene in cell proliferation and the induction and maintenance of cancer. SRSF3 was also found to be overexpressed in various cancer types, namely, respiratory, gastrointestinal, genitourinary, endocrine, and mesenchymal tissue-derived tumors (12, 13). Additionally, SRSF3 is upregulated in human ovarian cancer and its knockdown leads to apoptosis of cancer cell, which indicates SRSF3 expression may significantly correlate to survival and immune cell infiltration (14). However, the expression and clinical values such as overall survival of SRSF3 in human pan-cancer still remain largely unknown. In our study, we conducted and visualized the prognostic values of SRSF3 in human pan-cancer using different databases, namely, the TCGA and Kaplan–Meier Plotter. Our findings indicated that SRSF3 is a novel oncogene related to prognosis using bioinformatic analysis and *in vitro*/*vivo* experiment. SRSF3 may be a clinical therapeutic target for human pan-cancer. Moreover, it could serve as potential target for cancer treatment, particularly in certain low-expression cancers.

## Materials and Methods

### Data Collection and Processing

Pan-cancer sequencing data was obtained from The Cancer Genome Atlas (TCGA) database to analyze through the portal websites (15, 16). Corresponding clinical characteristics, namely, age of patients, sex, tumor, and clinical stages were also collected through the TCGA website. R package ‘rma’ was utilized in R (R version: 3.6.2) to filter data, delete missing and duplicated consequences and convert the whole data by log2(X + 0.001) (17). Additionally, the data extracted from the TCGA was used to perform tumor mutation burden (TMB) and microsatellite instability (MSI), which represents total mutation occurrence per million base pair and the quantity of insertion or deletion incident happened in duplicating sequences of genes (18–20).

### Clinical Specimens

Three of each kind of cancer tissues and paired normal tissues, namely, CHOL, COAD, ESCA, GBM, HNSC, LIHC, LUAD, LUSC, STAD, KICH, KIRP, and THCA were recruited by the Shanghai Tongji Hospital and Shanghai Tenth People’s Hospital of Tongji University. The clinicopathological details are presented as **Additional File 1: Supplementary Table 1**. Ethical approval for the study was granted by the Clinical Research Ethics Committee, Shanghai Tongji Hospital and Shanghai Tenth People’s Hospital of Tongji University.

### Gene Set Enrichment Analysis (GSEA)

GSEA was employed through the molecular signatures database, which offered hallmark gene sets to evaluate and forecast biological processes of normal and cancer samples. The parameters in the running software were set to replacement type selection phenotype, gene name selection, expression dataset selection gene cluster file and tag selection of phenotype: Cancer versus Normal. The replacement parameter was set to 1,000 times and a False discovery rate (FDR) less than 0.01 was considered as the cutoff. The ranking list consisted of gene symbol and log_2_-fold expression, which was in the declining sequence. Moreover, we conducted annotation, visualization as well as Kyoto Encyclopedia of Genes and Genomes (KEGG) by GOs and gene cascades examined with dataset.

### Cox Regression Analysis and Survival Analysis

The whole data obtained from the TCGA was used to performed Cox regression analysis utilizing R (R version: 3.6.2) to find out the correlation between the expression of SRSF3 and overall survival (OS)of the patients, disease-specific survival (DSS) and also disease-free interval (DFI) among 33 human pan-cancer types (21). The high and low expression of SRSF3 was used to divide patients into 2 groups and the Kaplan–Meier method was performed to establish the survival curses of patients among human pan-cancer types.

### Immune Cell Infiltration Enrichment

Immune cell infiltration calculation providing infiltration scores of various immune cells, which contain B cells naive, CD4^+^ T cells, CD4 memory activated/resting, CD8^+^ T cells, T cells follicular helper, T cells regulatory (Tregs), T cells gamma delta, monocytes, natural killer cells (NK cells), macrophages 0/1/2, neutrophils, eosinophils, mast cells activated/resting and dendritic cells, was performed using R packages (“ggplot2”, “ggpubr”, “ggExtra”, pFilter = 0.001). The scores of immune cell infiltration among human pan-cancer data obtained from the TCGA were calculated and archived through R software. In our study, the infiltration data were extracted and used to examine for correlation with the expression of SRSF3.

### Cell Culture and Transfection

Human colon adenocarcinoma and kidney carcinoma cell lines (SW480 and 786-O) were obtained from American Type Culture Collection. SW480 and 786-O cells were cultured in RPMI-1640 with 10% fetal bovine serum (FBS) and maintained in an incubator containing 5% CO_2_ at 37°C. 3 × 10^5^ of SW480 and 786-O cells were seeded in a six-well plate and incubated for 12 h for attachment. The cells were then transfected with the related reagents using Lipofectamine™ 3000 (Thermo Fisher Scientific, USA) according to the protocol of the manufacturer. The specific short hairpin RNAs (shRNAs) for SRSF3 (sh#1/2), relative negative control (NC; sh-NC), the pcDNA3.1-SRSF3 and empty vectors were all procured from WZ Biosciences Inc. (Shandong, China).

### Western Blotting

Cells were isolated and denatured in SDS buffer for total proteins. Total protein was separated by SDS-PAGE gel and transferred onto PVDF (polyvinylidene difluoride) membranes (Millipore, USA). After blocked in 5% non-fat milk for 1 h, membranes were incubated overnight at 4°C with the indicated primary antibodies, anti-SRSF3 (Abcam ab198291, 1:1,000 dilution), anti-Bcl-2, anti-Bcl-xl, anti-Mcl-1, (Abmart T40056 T40057 T40058, 1:1,000 dilution, Shanghai) and anti-GAPDH (Proteintech, #CL488-60004 1:5,000 dilution) antibodies, followed by incubation with secondary antibodies for 1 h at room temperature, and visualized by the ECL chemiluminescence reagent (Millipore, USA).

### Establishment of *In Vivo* Tumor Models

Nude mice (female, 4 weeks) were obtained from the Hubei Laboratory Animal Centre of Tongji University (Shanghai, China). SW480 and 786-O (Overexpression/Knockdown of SRSF3 and control) cells were injected subcutaneously (1 × 10^6^cells/100 µl PBS/mouse) into the subaxillary region of the nude mice to generate tumors with size of 60 mm^3^. A total number of 18 mice were randomly divided into 3 groups (n = 3 per group per cell type). After 21 days the mice were sacrificed and the tumors were removed and measured.

### Immunohistochemistry

The incubated slides were deparaffinized in xylene and then rehydrated with graded alcohol. Sequentially, each slide was covered with 10% goat serum in phosphate buffered saline for 10 min at room temperature and then incubated with SRSF3 polyclonal antibody (ProteinTech Group Cat no. 10916-1-AP, 1:100 dilution) as the primary antibody at 4° overnight. Goat Anti-Rabbit lgG (H+L) (Jackson Cat no.111035003) was used as the secondary antibody. All slides were counterstained with hematoxylin and the staining intensity was evaluated as integrated optical density (IOD) in the area of positive cells (intensity) or IOD per area of positive cells (IOD/area, mean intensity) using Image Pro Plus 6.0 software (Media Cybernetics, MD, USA). In each group, at least three random visual fields in three sequential sections per human tissue were evaluated.

### Statistics

The correlation among SRSF3 expression and the immune cell infiltration scores, TMB and MSI (as depicted previously) was assessed using the Spearman correlation test. Paired *t-*test was used to compare SRSF3 expression between groups either between tumor and normal tissues. If the data were not paired then the *t*-test was used to perform the analysis. *P*-values less than 0.05 represent statistical significance. All results were generated and visualized using ‘ggplot2’ and ‘forestplot’ R package.

## Results

### Evaluation of SRSF3 Expression in Different Cancer and Normal Tissues

**Figure 1:**
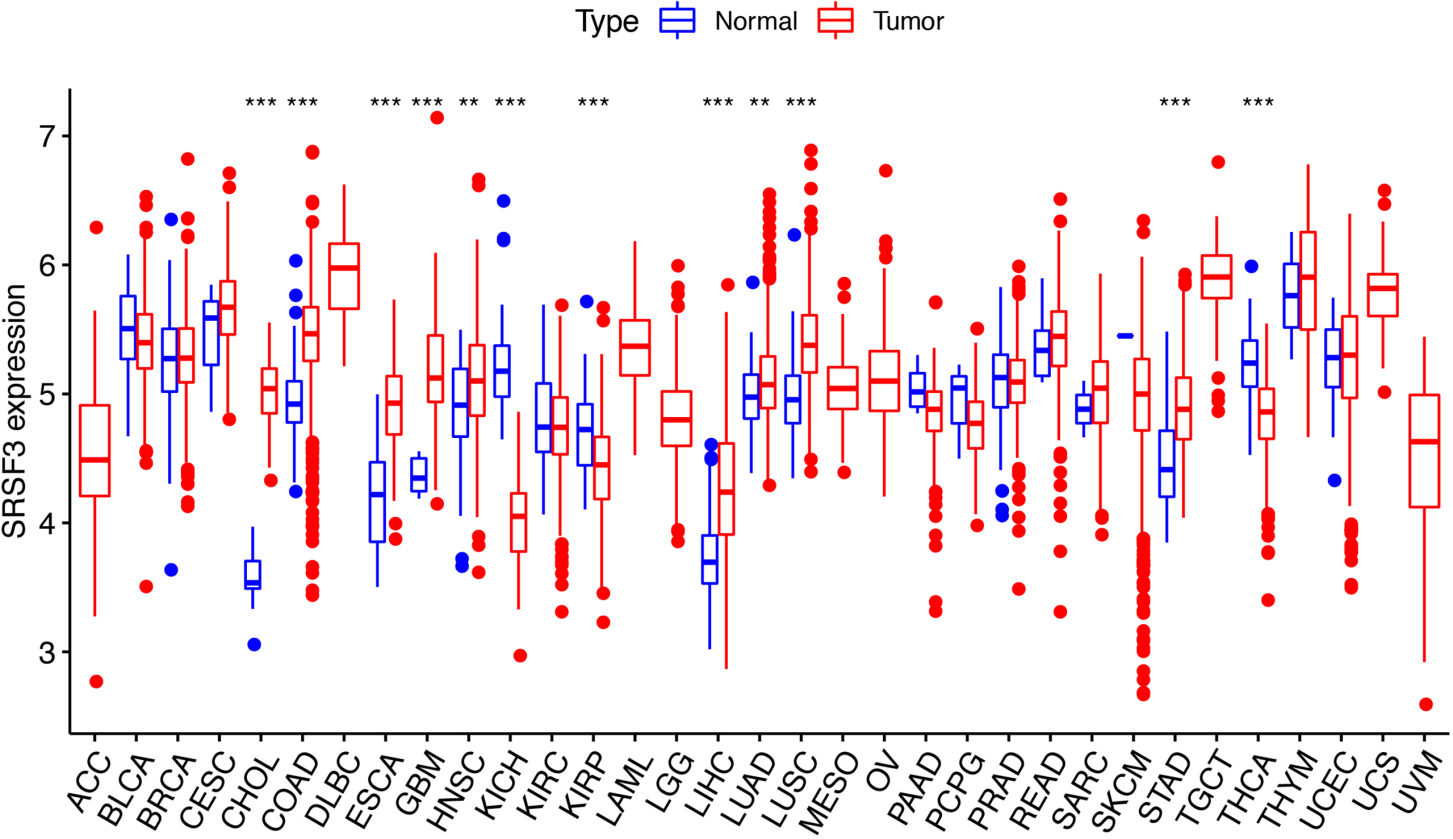
SRSF3 expression in cancers. Human SRSF3 expression level in 33 human pan-cancers from the TCGA database. *P < 0.05, **P < 0.01, ***P < 0.001.

**Figure 2:**
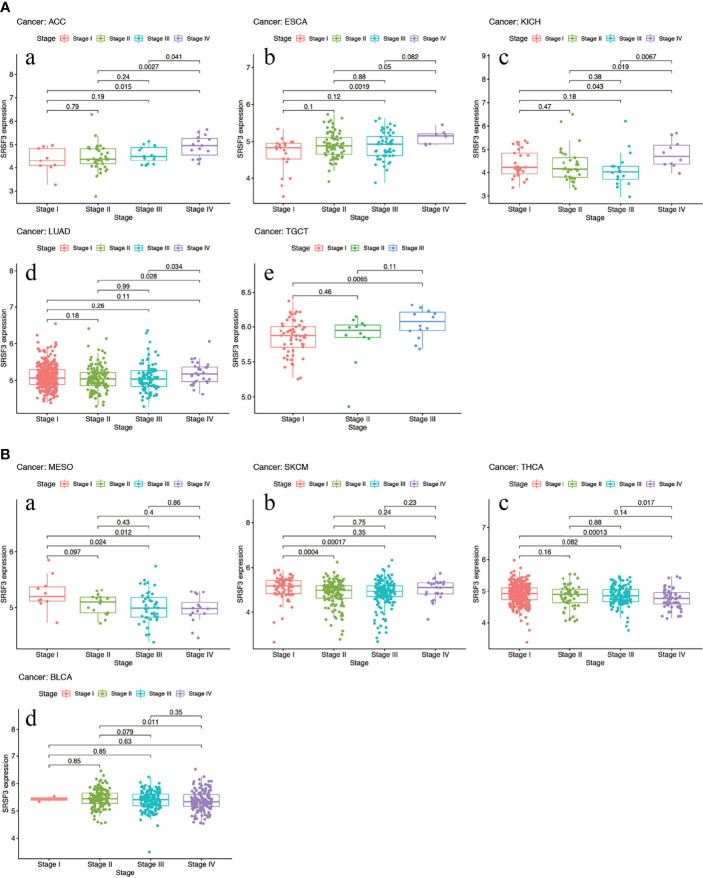
High expression of SRSF3 correlates with advanced TNM stage in human cancer. **(A)** Expression of SRSF3 is higher in more advanced TNM stage in ACC **(a)**, ESCA **(b)**, KICH **(c)**, LUAD **(d)** and TGCT **(e)**. **(B)** Expression level of SRSF3 is lower in more advanced TNM stage in BLCA **(a)**, MESO **(b)**, SKCM **(c)** and THCA **(d)**. *P <*0.05 is considered to be statistically significant.

The expression of SRSF3 in different tumor and normal tissue types were evaluated by utilizing the TCGA database and the results showed that the expression of SRSF3 was upregulated relative to normal tissue types in 9 out of 33 pan-cancers, namely, CHOL, COAD, ESCA, GBM, HNSC, LIHC, LUAD, LUSC, and STAD. Additionally, compared with normal tissue, the expression of SRSF3 was much lower in 3 out of 33 pan-cancers, namely, KICH, KIRP, and THCA (**Figure 1**). Moreover, the expression of SRSF3 was higher in more advanced Tumor Node Metastasis (TNM) in ACC, ESCA, KICH, LUAD, and TGCT. In contrast, SRSF3 expression was found to be lower in more advanced TNM stage in BLCA, MESO, SKCM, and THCA (**Figure 2**).

### The Connection Between SRSF3 Expression and Cancer Patient Prognosis

**Figure 3:**
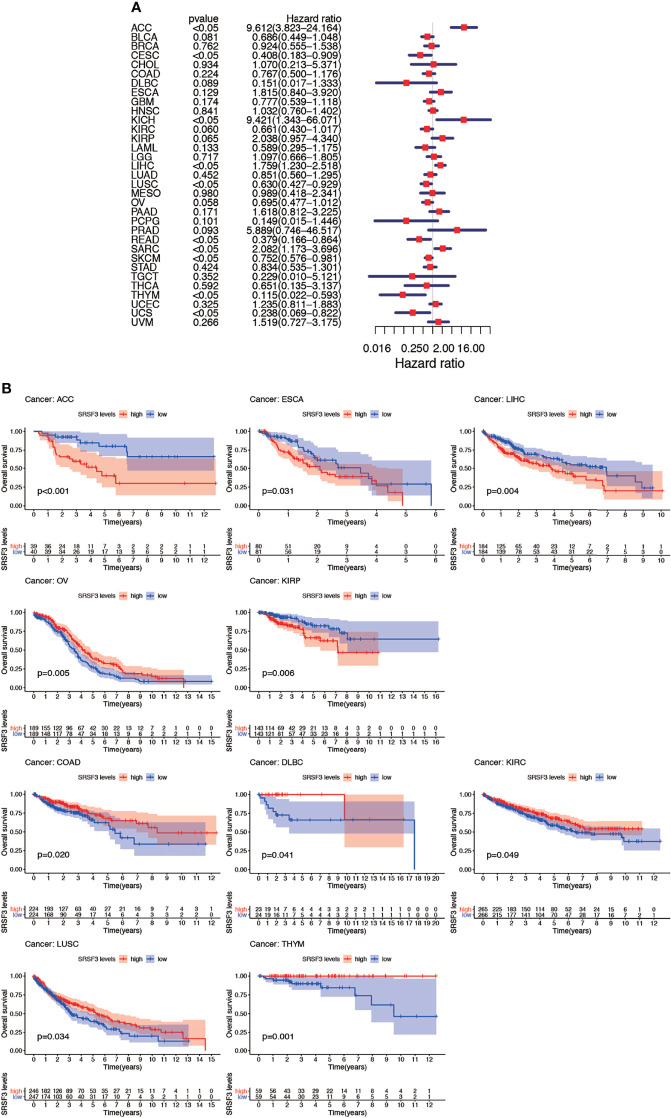
Association between expression levels of SRSF3 and OS in different tumors from the TCGA database. **(A)** Cox regression analysis showed that upregulated SRSF3 expression was significantly associated with worse prognosis in ACC, ESCA, KIRP, and LIHC cancer. Upregulated expression of SRSF3 was significantly associated with better prognosis in COAD, DLBC, KIRC, LUSC, OV, and THYM cancer. *P <*0.05 is considered to be statistically significant. **(B)** OS difference between high and low SRSF3 expression groups (divided by median expression values) in significant prognosis-related tumors from TCGA database. OS difference between groups in ACC. OS difference between groups in COAD. OS difference between groups in DLBC. OS difference between groups in ESCA. OS difference between groups in KIRC. OS difference between groups in KIRP. OS difference between groups in LIHC. OS difference between groups in LUSC. OS difference between groups in OV. OS difference between groups in THYM. *P* < 0.05 is considered to be statistically significant and dash lines represents 95% confidence interval.

The connection between the expression of SRSF3 and the outcome of patients with cancer were identified by utilizing the TCGA database. Interestingly, several cancer types presented a significant connection among prognosis of the patient and the expression of SRSF3 in cancers, namely, ACC, COAD, DLBC, ESCA, KIRC, KIRP, LIHC, LUSC, OV, and THYM. Additionally, the Kaplan–Meier plotter database was conducted so as to evaluate the relationship between SRSF3 expression and prognosis among cancer types. We found that the upregulated SRSF3 expression was significantly associated with ACC, ESCA, KIRP, and LIHC cancer. Nevertheless, the decreased expression of SRSF3 in COAD, DLBC, KIRC, LUSC, OV, and THYM cancer patients were found that got poorer prognosis (**Figure 3A**). The correlation between the respective expression level of SRSF3 and OS among 33 human pan-cancer types was examined through single variate Cox regression analysis using data obtained from the TCGA database. The results showed that the hazard ratios for SRSF3 were statistically significant for ACC, ESCA, KICH, LIHC, READ, SARC, SKCM, THYM, and UCS, among which SRSF3 had the highest effect in ACC (**Figure 3B**).

**Figure 4:**
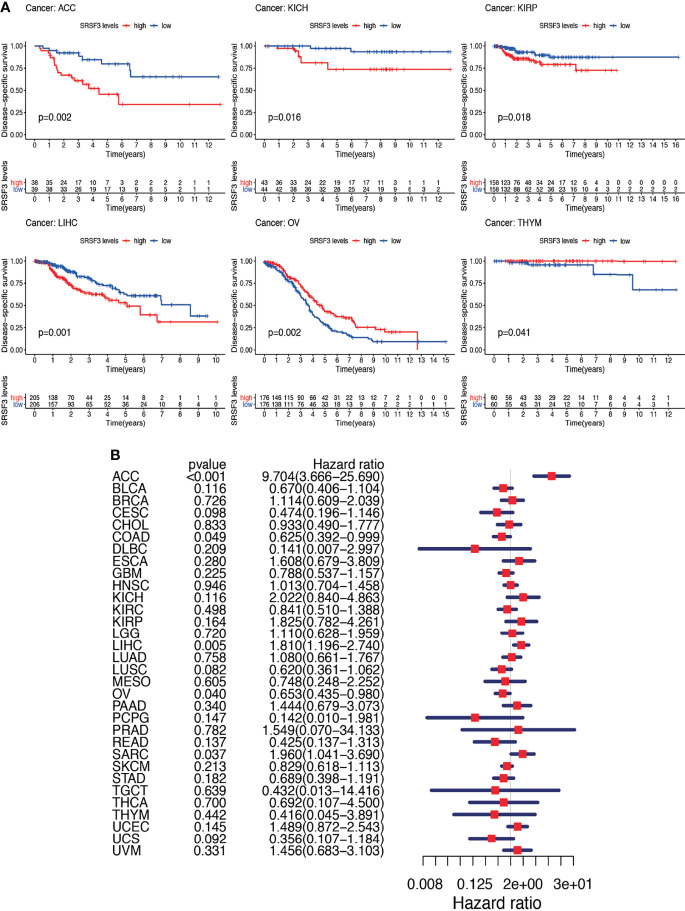
Between DSS and the expression of SRSF3 among human pan-cancer. **(A)** Association between expression levels of SRSF3 and DSS in different tumors from TCGA database. *P <*0.05 is considered to be statistically significant. **(B)** DSS difference between high and low SRSF3 expression groups (divided by median expression values) in significant prognosis-related tumors from TCGA database. DSS difference between groups in ACC. DSS difference between groups in KICH. DSS difference between groups in KIRP. DSS difference between groups in LIHC. DSS difference between groups in OV. DSS difference between groups in THYM. *P* < 0.05 is considered to be statistically significant and dash lines represents 95% confidence interval.

Nevertheless, the results of OS could be influenced by non-cancer according to deaths during the following period and thus the data from the TCGA were analyzed for correlation between DSS and the expression of SRSF3 among human pan-cancer. The consequences of the Cox regression analysis indicate familiar consequences to those correlating to OS (**Figure 4A**). Patients with high SRSF3 expression level in the majority of cancer types indicated a worse prognosis comparing with the low expression groups in the subsequent survival analysis (**Figure 4B**).

**Figure 5:**
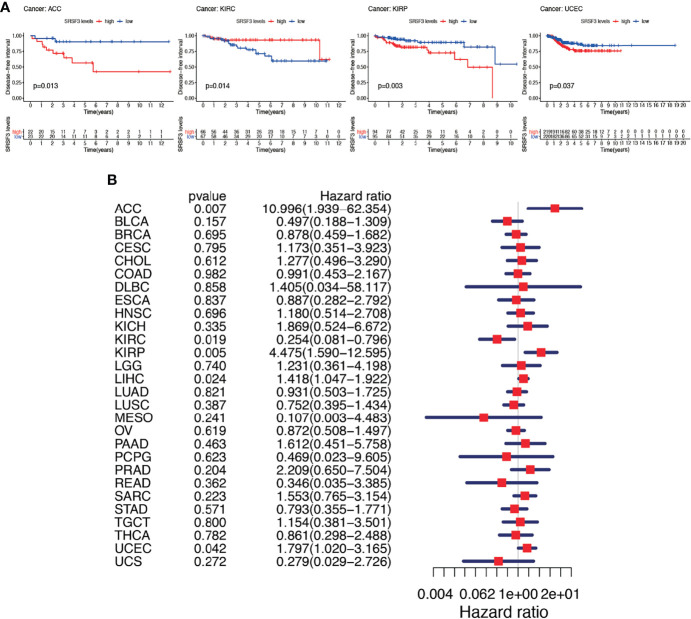
Between DFI and the expression of SRSF3 among human pan-cancer. **(A)** DFI difference between high and low SRSF3 expression groups (divided by median expression values) in significant prognosis-related tumors from TCGA database. DFI difference between groups in ACC. DFI difference between groups in KIRC. DFI difference between groups in KIRP. DFI difference between groups in UCEC. **(B)** Association between expression levels of SRSF3 and DFI in different tumors from TCGA database. P < 0.05 is considered to be statistically significant.

Subsequently, Cox regression analysis was performed again to examine the correlation between the expression of SRSF3 and DFI. The consequence of DFI showed that hazard ratios were found to be significant in ACC, KIRC, KIRP, LIHC, and UCEC (**Figure 5A**). Patients were divided into two groups depending on the median expression of SRSF3 and there showed statistical difference between high and low expression SRSF3 groups in survival differences. Among previously mentioned cancer types, patients with high expression of SRSF3 showed early recurrence tumorectomy post-operatively except KIRC. Additionally, the significance of SRSF3 expression in cancer progression was emphasized by the gap over 3 years between survival times (according to DFI) of different patient group with high and low expression of SRSF3 (**Figure 5B**).

### SRSF3 May Regulate the Tumor Immune Microenvironment Through Affecting Immune Infiltration Among 33 Human Pan-Cancer Types

SRSF3 was reported that its expression level has multi-biological function in tumor cells proliferation and metabolism, metastasis, apoptosis, cell cycle and also immune response. Therefore, the correlation between SRSF3 expression and the infiltration level of various immune cells among 33 human pan-cancer types was examined to assess whether SRSF3 can influence immune microenvironment of tumors or not. The results shown in **Supplementary Figure 1** indicated that there did exist significant correlation between SRSF3 expression and several tumors types, namely, BLCA, BRCA, ESCA, COAD, DLBC, HNSC, KIRC, KIRP, LAML, LIHC, LUAD, LUSC, OV, PRAD, READ, SKCM, STAD, THCA, THYM, UCEC, and UVM. These corresponding linear regression results showed that the high expression level of SRSF3 was associated with a potential upregulated infiltration level by various immune cells.

### SRSF3 is Linked With the TMB and MSI in Certain Cancers

The consequences of TMB and MSI were considered as valid prognostic biomarkers and the reaction indicator of immune treatment among various types of cancers and therefore, the relationship of TMB and MSI with the expression of SRSF3 was conducted to investigate the association between SRSF3 activity and mutation among 33 human pan-cancer types. The consequence showed that the correlation between SRSF3 expression level and TMB exists significance (*P <*0.05) in 15 out of 33 cancer types, namely, ACC, BLCA, BRCA, COAD, HNSC, LGG, LUAD, LUSC, OV, READ, STAD, TGCT, THCA, THYM, and UCEC, of which STAD had the highest coefficients while THYM had the lowest coefficients (**Supplementary Figure 2A**). The consequence of coefficient analysis indicated that SRSF3 expression had positive correlation concerning high mutation status in ACC, BLCA, BRCA, COAD, HNSC, LGG, LUAD, LUCS, OV, READ, STAD, TGCT, and UCEC while low in THCA and THYM particularly. At the meantime, correlation of SRSF3 expression with MSI was also examined among 33 human pan-cancer types, of which 11 out of 33, namely, BRCA, CESC, ESCA, GBM, HNSC, LUSC, READ, SARC, STAD, THCA, and UCEC, achieved statistical significance (*P <*0.05) in **Supplementary Figure 2B**. According to the results, HNSC, READ, STAD, and UCEC had the highest coefficients, which indicated a positive correlation between SRSF3 expression and MSI, while CESC, GBM, and LUSC showed a negative correlation between SRSF3 expression and MSI. Particularly, CESC and GBM had the lowest coefficient values among these cancer types. Comparing among 33 human pan-cancer types, it is remarkable that BRCA, HNSC, and UCEC showed relatively high coefficient for correlation while LUSC and THCA were in contrast both in TMB and MSI.

### The Correlation Between the Expression of SRSF3 and the Expression of Some Immune Checkpoint Genes in Cancer

We evaluated the relationship between SRSF3 expression and immune checkpoint gene expression by obtaining mRNA sequence database, and analyzed the relationship between SRSF3 and immune response. Genes that are regarded as checkpoints in the immune response are often closely related to the immune response and can be used to predict the correlation with gene co-expression. According to the correlation analysis between SRSF3 and immune checkpoint gene expression, it can be found that SRSF3 is closely related to TNFSF14, TNFRSF14, TNFRSF25, CD276, NRP1, VSIR in a variety of malignant tumors (*P <*0.05). SRSF3 is closely related to tumor necrosis factor (TNF)-related ligands and receptors, suggesting that SRSF3 may play an important role in tumor immunity. SRSF3 and CD276 have significant co-expression (*P <*0.05), and CD276 (B7-H3), as a possible immune checkpoint molecule, is predicted by researchers to become one of the most promising tumor immunotherapy targets (22). This suggests that SRSF3 plays an important role in a variety of tumor immunity. In addition, in KICH and LIHC, SRSF3 is significantly co-expressed with more immune checkpoint genes, indicating that SRSF3 is easier to regulate tumor immune response by regulating immune checkpoint activity in these three malignant tumors. But surprisingly, SRSF3 is negatively correlated with most immune checkpoints of tumors such as BRCA, GBM, LGG, and TGCT, suggesting that SRSF3 may play a negative regulatory role in tumor immunity (**Figure S2C**).

### Explore SRSF3 Expression in Clinical Specimen and its Functions During Tumor Progression *In Vitro* and *In Vivo*

**Figure 6:**
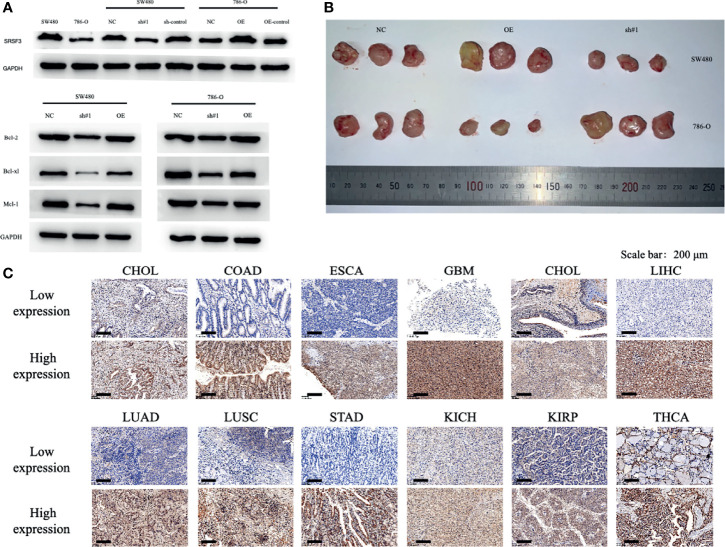
Functional characterization of SRSF3 *in vitro*/*vivo* and representative of immunohistochemistry staining of SRSF3 in normal and tumor tissues. **(A)** Western blot evaluations were used to evaluate SRSF3 expression in SW480 and 786-O and confirm the efficacy of transfection. **(B)** tumor size in mice of NC group, OE SRSF3 group and sh#1 SRSF3 group. **(C)** Representative western blots of apoptosis related protein expression in SW480 and 786-O cells after transfection. GAPDH as the loading control. High SRSF3 expression in CHOL, COAD, ESCA, GBM, HNSC, LIHC, LUAD, LUSC and STAD tumor tissues. Low SRSF3 expression in KICH, KIRP and THCA tumor tissues. Scale bar: 200 µm.

The SRSF3 expressions in CHOL, COAD, ESCA, GBM, HNSC, LIHC, LUAD, LUSC, STAD, KICH, KIRP, and THCA normal and tumor tissues were validated by immunohistochemistry and the staining were in accordance with the results from the TCGA database. The results of IOD were significantly statistical difference among 12 cancer types between normal and tumor tissues by immunohistochemistry staining. To determine the role of SRSF3 during tumor progression, we performed loss and gain-of-function assays using lentivirus-mediated knockdown and overexpression systems in SW480 and 786-O cells *in vitro* and *in vivo*. The expression levels of SRSF3 in SW480 and 786-O cells were verified by western blot. Additionally, we investigated the potential mechanism of SRSF3 in cancer cell apoptosis in SW480 and 786-O cells. Western blotting showed that SRSF3 knockdown decrease the anti-apoptotic proteins expression, namely, Bcl-2, Bcl-xl, and Mcl-1 in SW480 cells while increase in 786-O cells. Collectively, these results indicated that higher SRSF3 expression accelerated the growth of cells in SW780 but inhibit in 786-O cells, which were consistent to the results of IHC. Finally, a xenograft tumor experiments were conducted to evaluate the effect of SRSF3 on different types of tumors using knockdown/overexpression level of SRSF3 tumor cells. SW480 and 786-O cells that over or knockdown expressions of SRSF3 were subcutaneously injected into the nude mice, correspondingly. Twenty one days later, SRSF3 knockdown in SW480 and overexpressed in 786-O tumors were significantly smaller and weighed less than control (**Figure 6**).

### GSEA Analysis

We analyzed the relevant signal transduction pathways of SRSF3 in 33 tumors through GSEA to identify the pathways that are differentially activated in various malignant tumors when SRSF3 is highly expressed. According to the results of GO enrichment analysis, it showed that GO terms rich in high SRSF3 phenotype mainly included DNA replication and regulating cell cycle (**Supplementary Figure 3A**). The high SRSF3 phenotype enriches multiple KEGG pathways related to cancer, such as MESO and OV. The low SRSF3 phenotype is enriched in most KEGG pathways, such as ACC, CHOL, GBM, LGG, LUSC, TGCT, and READ. These signaling pathways mainly include RIG-I-like receptors, drug metabolism, and hormone biosynthesis. In STAD and THYM, the high or low SRSF3 phenotype has enriched signal pathways related to cancer (**Supplementary Figure 3B**).

## Discussion

Evidence indicates that SRSF3 as a general splicing factor has a variety of functions from affecting genome stability to transcription and also RNA processing and protein translation (23–26). Moreover, early study in nude mice reported that higher expression level of SRSF3 promotes cell immortalization and transformation, which is needed for cancer induction and maintenance (12, 14, 27, 28). Additionally, SRSF3 is upregulated in a variety of tumors and acts as a proto-oncogene. Previous researches have shown that SRSF3 expression was associated with cancer progression and correlated significantly with worse survival and shorter disease-free survival. However, there is no study exploring the oncogenic of role SRSF3 comprehensively in human pan-cancer. Our bioinformatic consequences have shown that SRSF3 was overexpressed among 33 human pan-cancer types when comparing with corresponding normal tissues. The high expression level of SRSF3 was associated to worse OS and death-specific survival among pan-cancer types. A correlation with progression of disease was recognized for ACC, KIRP, LIHC, and UCEC, for which patients with high expression level of SRSF3 triggered from early tumor recurrence and these results were validated by immunohistochemistry (*P <*0.05). However, KIRC was an exception to this situation. The results of SRSF3 were reported systemically in human pan-cancer first by us and it may indicate that SRSF3 may act as an oncogenic driver and an interesting biomarker for tumor monitoring in further research (29, 30).

Dysregulation of SRSF3 is correlated with carcinogenesis in pan-cancer and we chose typical cell lines, namely, SW480 and 786-O cells to perform *in vitro* and *in vivo* experiments to verify it. Functional assay showed that SRSF3 is correlated to cell apoptosis therefore affecting tumor progression. Additionally, SRSF3 knockdown SRSF3 expression in SW480 significantly inhibits tumor growth in nude mice while it has opposite effects in 786-O, which is consistent to the result of clinical specimen by IHC staining and previous researches (10). Collectively, these data present that SRSF3 may serve as an oncogene and a potential prognostic biomarker in human 33 pan cancer.

Although SRSF3 has not been well studied in immune-oncology, several researches have proposed that the expression of SRSF3 was related to human immunity. The results of correlation analysis have shown that SRSF3 expression was clearly related to immune cells infiltration among human pan-cancers and particularly in BRCA and THYM had the highest coefficients values with immune cells infiltration (31, 32). Among various immune cell types in our study, T cells counted the highest coefficients values. Interestingly, SRSF3 expression was also linked to upregulation of certain specific immune checkpoint genes among different cancer types, such as KICH, LIHC, and SRSF3, which may result in immune cells differentiation and polarization. Wu et al. also demonstrated a function for SRSF3 in PD-1 mRNA extranuclear transport in T cells (33). Notwithstanding, due to the multiplicity of interactions of SRSF3 and its multi-functional association, it can promote tumor alterations *via* other unknown mechanisms.

Therefore, understanding tumor microenvironment, including immune cell infiltration can possibly help unveil the mechanisms behind tumor development. Subsequently, early studies have shown that TMB and MSI were linked to medicine reactions of patients, in particular for those medicines targeting to immune checkpoint inhibitors. The associations of SRSF3 expression level and TMB and MSI among human pan-cancers were demonstrated and the results have shown that SRSF3 could act as an extra indicator for immune treatment assessment of cancer patients after management (34, 35). According to published studies, MSI is now considered as an indicator for distinguishing the cancer type in patients with COAD and moreover, those COAD patients with high MSI indicated better checkpoint inhibitor feedbacks and survival from low to advanced TNM or clinical stages (36). In our study, both TMB and MSI in COAD were positively linked to SRSF3 expression, which are consistent to previous results but only TMB achieved significant difference. It supports our proposition that SRSF3 could be an additional indicator for possible medicine reactions for both TMB and MSI in BRCA, HNSC, and UCEC were also positively linked to SRSF3 expression (37, 38).

Even though our results provided useful information of the association of SRSF3 expression level with tumorigenesis and regulation of the immune environment in cancers and additionally the OS, some limitations existed in this study. First, this study conducted a bioinformatic analysis of SRSF3 and patient survival *via* data from the TCGA database and though it was confirmed by *in vitro* and *in vivo* experiments it was lacking experiments for verifying immune cells infiltration, which needed further studies. Our results of SRSF3 only depended on the mRNA levels presented *via* database but the functional proteins were also not reflected for protein activity could be affected by multi-factors such as post-transcription modification or proteolysis. Further mechanistic studies on SRSF3 at the cellular and molecular levels could help to explain the role of SRSF3. Second, in spite of the result that SRSF3 expression correlates with various immune cells infiltration and overall survival in pan-cancers of patients, we still could not confirm that SRSF3 affects survival of patients *via* immune infiltration, and therefore further research could focus on this field and find out the mechanism. Third, the association between SRSF3 expression with TMB and MSI genes were lacking of *in vivo*/*vitro* data to find out and explain any intrinsic mechanism.

### Conclusion

We have demonstrated the correlation of expression level of SRSF3 and overall survival across 33 human pan cancer types. SRSF3 is expressed in various cancers and its high expression correlated to poor survival and disease progression, particularly for ACC, KIRP, and UCEC. Additionally, SRSF3 expression was associated with immune cells infiltration, check point gene expressionand immune treatment indicators. In summary, SRSF3 expression can be considered as a prognostic biomarker in pan-cancer and therapeutic evaluation.

## Data Availability Statement

The datasets presented in this study can be found in online repositories. The names of the repository/repositories and accession number(s) can be found in the article/**Supplementary Material**.

## Ethics Statement

The studies involving human participants were reviewed and approved by the Shanghai Tongji Hospital:2021-KYSB-061. The patients/participants provided their written informed consent to participate in this study. Written informed consent was obtained from the individual(s) for the publication of any potentially identifiable images or data included in this article.

## Author Contributions

ZL performed the data analyses, *in vivo/vitro* experiments and wrote the manuscript. HH validated the data and revised the visualization of data. XW and TY took charge of the investigation of patients’ characteristic, and collected clinical samples. FX and HZ provided technical assistance and revised the part of methodology. The corresponding author, AS, supervised the project and reviewed the manuscript, YY designed this study and offered the financial support (funded by National Natural Science Foundation of Shanghai: Grant No. 21ZR1458500 and by Shanghai Tongji Hospital: ITJ(ZD)2004) for the project.

## Conflict of Interest

The authors declare that the research was conducted in the absence of any commercial or financial relationships that could be construed as a potential conflict of interest.

## Publisher’s Note

All claims expressed in this article are solely those of the authors and do not necessarily represent those of their affiliated organizations, or those of the publisher, the editors and the reviewers. Any product that may be evaluated in this article, or claim that may be made by its manufacturer, is not guaranteed or endorsed by the publisher.

## Supplementary Material

Supplementary Figure 1 Estimate correlation between SRSF3 expression level and infiltration of various immune cells (B cells naive, CD4^+^ T cells, CD4 memory activated/resting, CD8^+^ T cells, T cells follicular helper, T cells regulatory (Tregs), T cells gamma delta, monocytes, natural killer cells (NK cells), macrophages 0/1/2, neutrophils, eosinophils, mast cells activated/resting and dendritic cells).

Supplementary Figure 2 Relation between TMB, MSI and SRSF3 expression levels in different tumors from TCGA database. **(A)** Correlation between TMB and SRSF3 expression. **(B)** Correlation between MSI and SRSF3 expression. Spearman correlation test was conducted and *P* < 0.05 is considered to be statistically significant. **(C)** Correlation between SRSF3 expression levels and recognized immune checkpoints’ expression in different tumors from TCGA database. The underlying triangle in each tile represents coefficients calculated through Pearson’s correlation test, and the upper triangle represents log10 transformed *P* -value. **P* < 0.05, ***P <*0.01, ****P* < 0.001.

Supplementary Figure 3 Functional and pathway enrichment analysis. **(A)** KEGG pathways; **(B)** GO pathways.

## Abbreviations

SRSF3, Serine-rich splicing factor 3; ACC, Adrenocortical carcinoma; BLCA, Bladder Urothelial Carcinoma; BRCA, Breast invasive carcinoma; CESC, Cervical squamous cell carcinoma and endocervical adenocarcinoma; CHOL, Cholangiocarcinoma; COAD, Colon adenocarcinoma; DLBC, Lymphoid Neoplasm Diffuse Large B-cell Lymphoma; ESCA, Esophageal carcinoma; GBM, Glioblastoma multiforme; HNSC, Head and Neck squamous cell carcinoma; KICH, Kidney Chromophobe; KIRC, Kidney renal clear cell carcinoma; KIRP, Kidney renal papillary cell carcinoma; LAML, Acute Myeloid Leukemia; LGG, Brain Lower Grade Glioma; LIHC, Liver hepatocellular carcinoma; LUAD, Lung adenocarcinoma; LUSC, Lung squamous cell carcinoma; MESO, Mesothelioma; OV, Ovarian serous cystadenocarcinoma; PAAD, Pancreatic adenocarcinoma; PCPG, Pheochromocytoma and Paraganglioma; PRAD, Prostate adenocarcinoma; READ, Rectum adenocarcinoma; SARC, Sarcoma; SKCM, Skin Cutaneous Melanoma; STAD, Stomach adenocarcinoma; STES, Stomach and Esophageal carcinoma; TGCT, Testicular Germ Cell Tumors; THCA, Thyroid carcinoma; THYM, Thymoma; UCEC, Uterine Corpus Endometrial Carcinoma; UCS, Uterine Carcinosarcoma; UVM, Uveal Melanoma; TMB, tumor mutation burden; OS, Overall Survival; DSS, Disease-specific survival; DFI, disease-free interval; GSEA, Gene set enrichment analysis; KEGG, Kyoto Encyclopedia of Genes and Genomes; GO, Gene ontology.
