# Supplementary figures and images for: SRSF3 Expression Serves as a Potential Biomarker for Prognostic and Immune Response in Pan-Cancer
Source: Front Oncol. 2022 Apr 14;12:808530. doi: 10.3389/fonc.2022.808530 (PMC9047863; doi:10.3389/fonc.2022.808530)

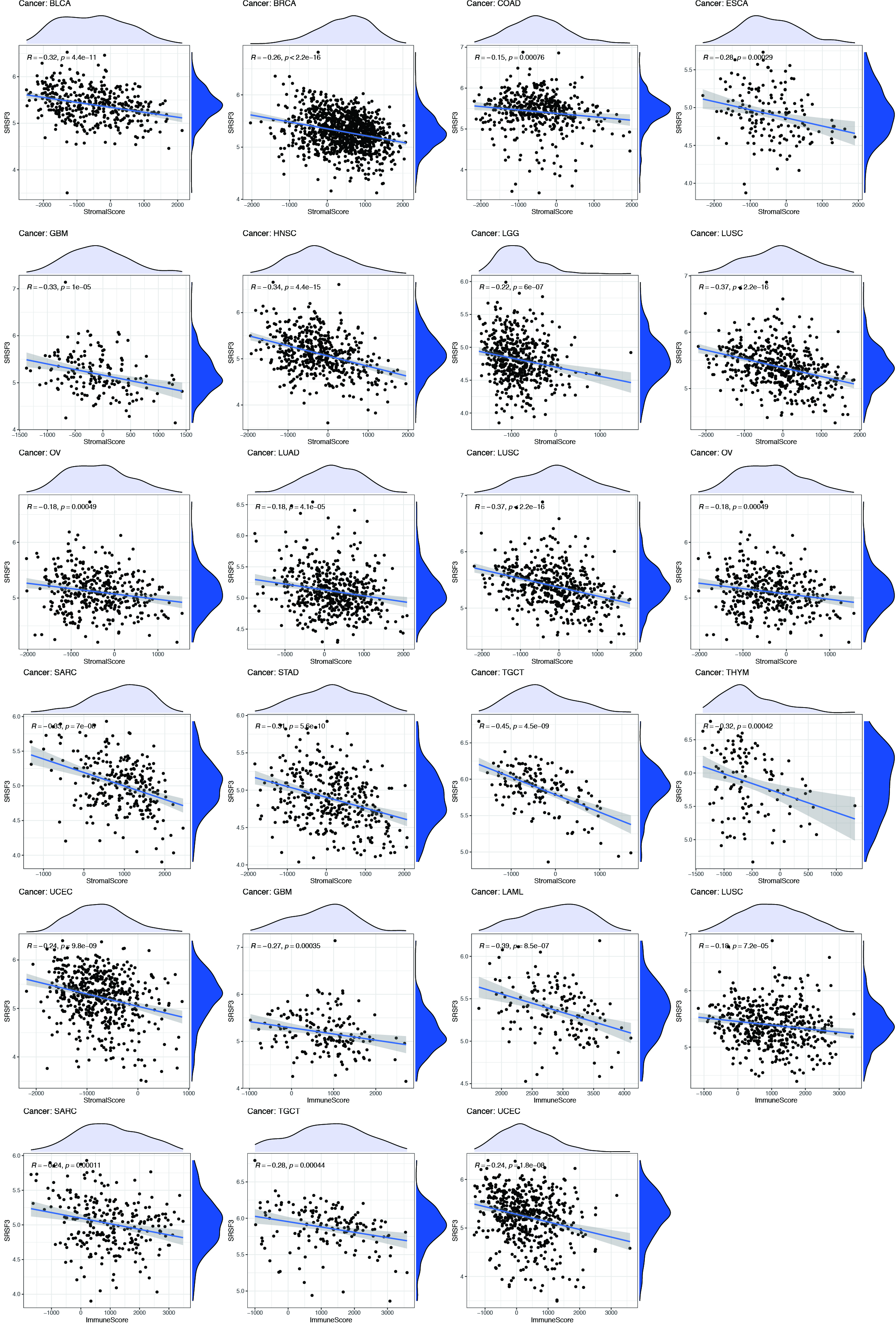

Supplement: Supplementary Figure 1 — Estimate correlation between SRSF3 expression level and infiltration of various immune cells (B cells naive, CD4+ T cells, CD4 memory activated/resting, CD8+ T cells, T cells follicular helper, T cells regulatory (Tregs), T cells gamma delta, monocytes, natural killer cells (NK cells), macrophages 0/1/2, neutrophils, eosinophils, mast cells activated/resting and dendritic cells). [file Image_1.jpeg]

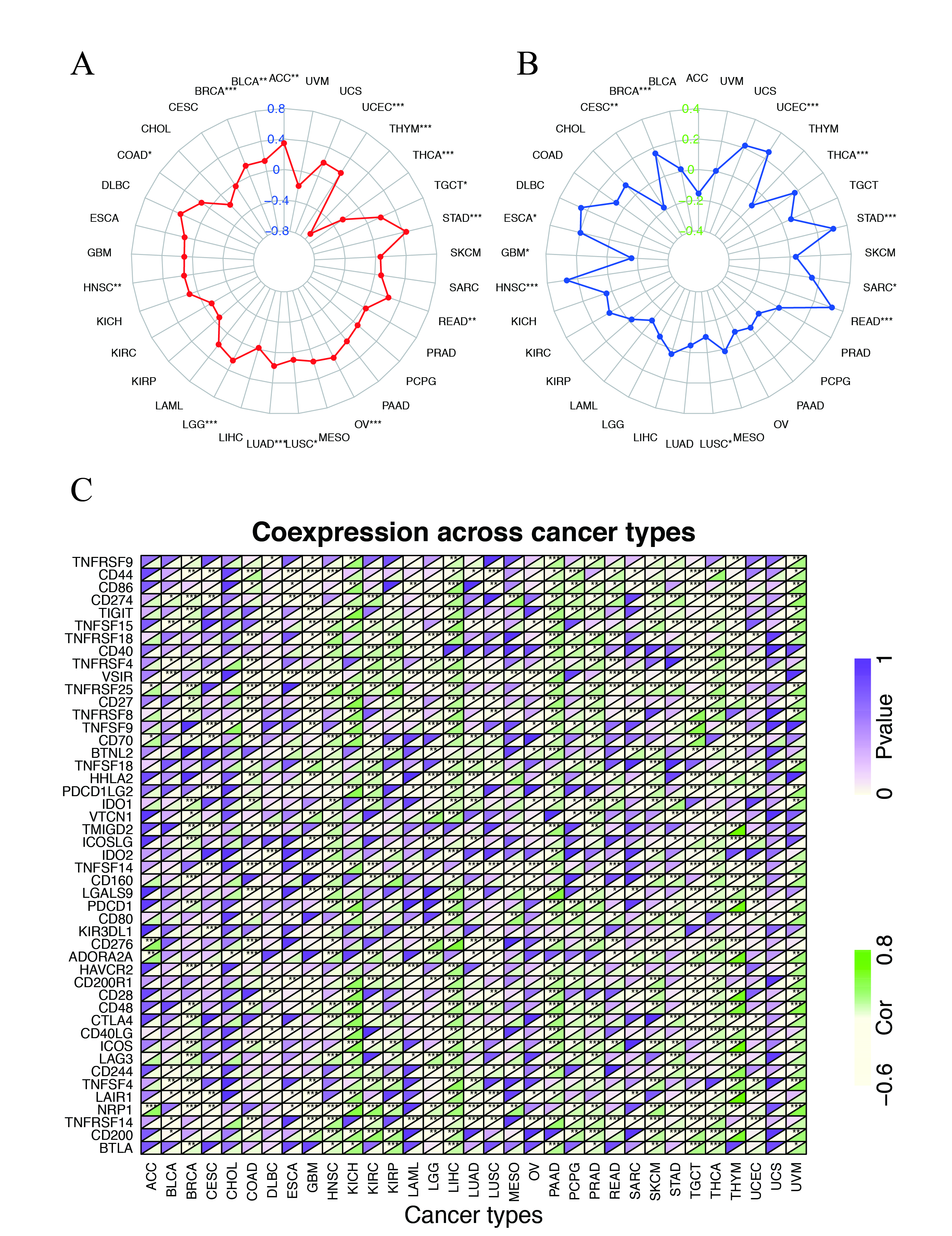

Supplement: Supplementary Figure 2 — Relation between TMB, MSI and SRSF3 expression levels in different tumors from TCGA database. (A) Correlation between TMB and SRSF3 expression. (B) Correlation between MSI and SRSF3 expression. Spearman correlation test was conducted and P < 0.05 is considered to be statistically significant. (C) Correlation between SRSF3 expression levels and recognized immune checkpoints’ expression in different tumors from TCGA database. The underlying triangle in each tile represents coefficients calculated through Pearson’s correlation test, and the upper triangle represents log10 transformed P -value. *P < 0.05, **P <0.01, ***P < 0.001. [file Image_2.jpeg]

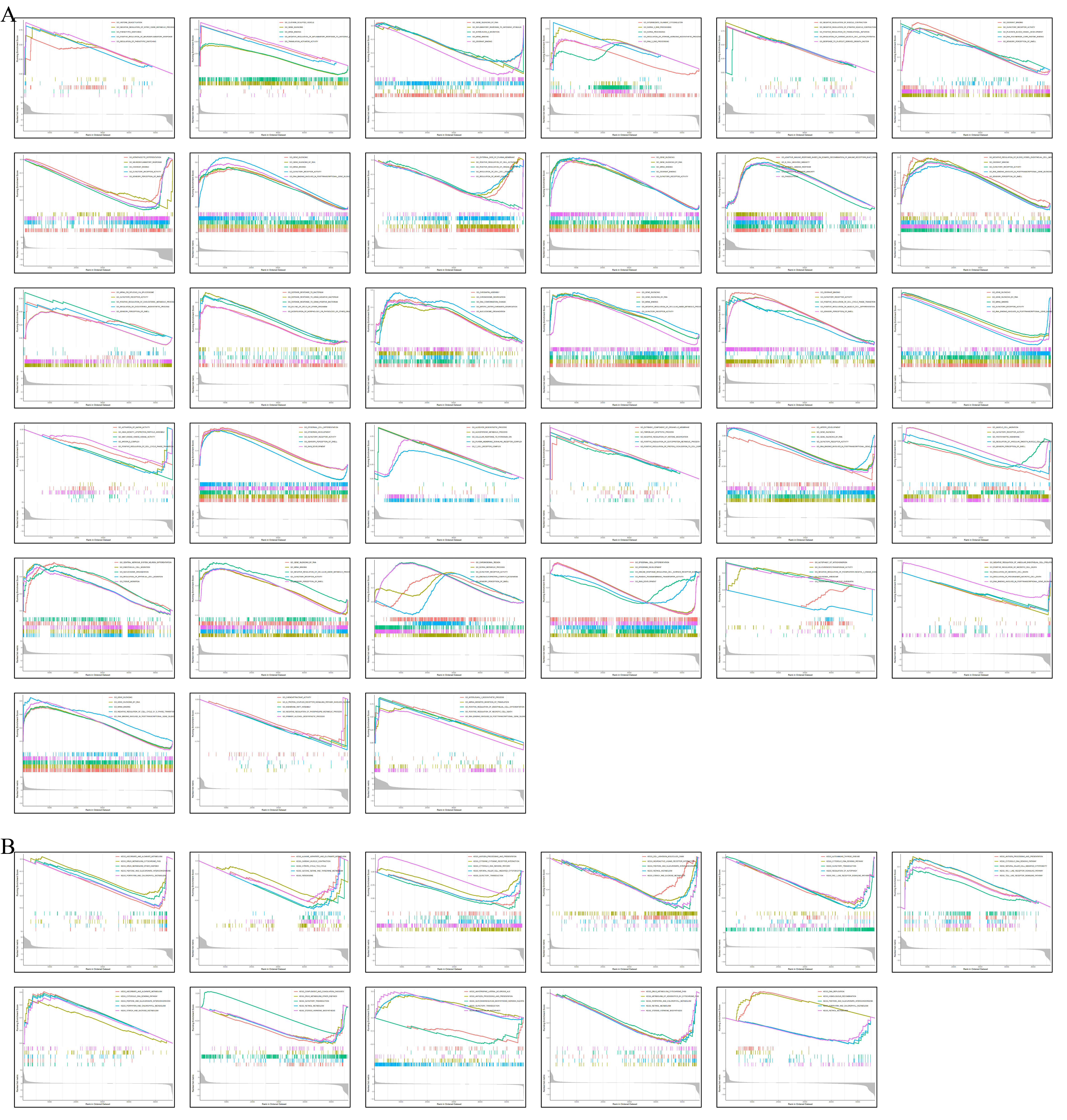

Supplement: Supplementary Figure 3 — Functional and pathway enrichment analysis. (A) KEGG pathways; (B) GO pathways. [file Image_3.jpeg]
